# Risk Profile of High-grade Cervical Lesions and Cervical Cancer Considering the Combination of Cytology, HPV Genotype, and Age among Women Undergoing Colposcopy

**DOI:** 10.1055/s-0043-1772483

**Published:** 2023-11-29

**Authors:** Júlio César Possati-Resende, Thais Zilles Fritsch, Karen Cristina Borba Souza

**Affiliations:** 1 Prevention Department, Hopital do Câncer de Barretos, SP, Brazil.; 2 Research and Education Institute, Hospital do Câncer de Barretos, Barretos, SP, Brazil.

**Keywords:** squamous intraepithelial lesions of the cervix, uterine cervical neoplasms, papillomavirus infections, colposcopy, risk, lesão escamosa intraepitelial cervical, neoplasias do colo do útero, infecções por papillomavirus, colposcopia, risco

## Abstract

**Objective** The present study aims to establish a risk profile for high-grade cervical lesions and cervical cancer (CIN2 + ) in women undergoing colposcopy at the Hospital do Câncer de Barretos, through the analysis of Human Papillomavirus (HPV) infection, cervical cytology, and patient's age.

**Methods** Retrospective cross-sectional study based on a computerized database of women aged ≥ 18 years old who underwent colposcopy at the Prevention Department of the Hospital do Câncer de Barretos from 2017 to 2019.

**Results** A total of 3,411 women were included, 58.0% were positive for high-risk-HPV test, with a higher prevalence of CIN2+ for HPV16 (30.3%) and other HPV (45.0%). Cytological findings that suggest invasive cervical cancer (squamous cells or adenocarcinoma), regardless of the status of HPV test, showed 100% diagnosis of CIN2 + , while atypias that suggest high-grade lesions, HSIL and ASC-H, positive for HPV test, showed in 86 and 55.2%, respectively, diagnosis of CIN2 + . ASC-H cytological results among women aged > 40 years old and negative HPV were mainly associated with benign findings. We observed that ≤ CIN1 has a higher prevalence among older women with negative HPV, while for high-grade lesions there is an increase among young women HPV16- and/or 18-positive. In cancer diagnosis, we observed a predominance of HPV 16/18 regardless of the age group.

**Conclusion** The highest risks of precursor lesions and cervical cancer were found among women with positive HPV 16/18 tests and severe cytological atypia in population screening tests. In addition, cytological findings of ASC-H HPV negative in women > 40 years old usually represent benign findings in histological investigation.

## Resumo

**Objetivo** Estabelecer um perfil de risco de lesões intraepiteliais de alto grau e câncer do colo do útero (NIC2 + ) em mulheres submetidas a colposcopia considerando-se a infecção pelo papilomavírus humano (HPV), citologia cervical e idade.

**Métodos** Estudo retrospectivo transversal em banco de dados informatizado de mulheres com idade ≥ 18 anos que realizaram colposcopia no departamento de Prevenção de Câncer no Hospital do Câncer de Barretos/SP no período de 2017 a 2019.

**Resultados** Foram incluídas 3.411 mulheres, sendo 58,0% positivas para HPV de alto risco, e maior prevalência de NIC2+ para HPV16 (30,3%) e outros HPV (45,0%). Resultados citológicos sugestivos de lesões invasivas (epidermoide ou adenocarcinoma), independente do teste de HPV, apresentaram 100% de diagnóstico NIC2 + , enquanto atipias sugestivas de lesões de alto grau, HSIL e ASC-H, associados a HPV positivo, apresentaram 86 e 55,2%, respectivamente. Resultados citológicos de ASC-H entre mulheres > 40 anos e HPV negativo foram associados principalmente a achados benignos. Observamos que ≤ NIC1 apresenta uma maior prevalência entre mulheres mais velhas com HPV negativo, enquanto para lesões de alto grau, há um aumento entre mulheres mais jovens positivas para HPV16/18. Para diagnóstico de câncer, observamos que há um predomínio de HPV16/18 independente da faixa etária.

**Conclusão** Foi identificado maior risco de lesões precursoras e câncer entre mulheres com HPV 16/18 positivo e atipias citológicas graves em testes de rastreio populacional. Além disso, resultados citológicos de ASC-H quando associados a HPV negativo com idade > 40 anos habitualmente representam achados benignos em investigação histológica.

## Introduction

Cervical cancer is one of the most frequent types of cancer among women worldwide, being the fourth in incidence and mortality, with an estimated 604,127 new cases in 2020. 1 Among the countries with the highest incidence of this disease, Brazil is fourth (17,743) in absolute numbers after India, China, and Indonesia. 1 2 According to data from the National Cancer Institute (INCA, in the Portuguese acronym), cervical cancer is the third most common cancer among Brazilian women (7.0%). 3

Persistent infection by human papillomavirus (HPV) is the leading cause of cervical cancer, with 14 types considered oncogenic (hrHPV). Types 16 and 18 together are responsible for ∼ 70% of squamous cell carcinomas, followed by types 31, 33, 45, 52, and 58. 4 5 6

The early detection of precursor lesions through population screening programs and the expansion of HPV vaccination coverage can minimize the impact of cervical cancer. 7 8 In Brazil, cervical cytology is recommended for women aged between 25 and 64 years old. Tests for the detection of HPV are not available for women assisted by the public health system; however, regional strategies and some population-based studies have been used. 9 10 11 12

The initiatives to implement molecular tests in population screening are justified because there is evidence that the presence of hrHPV infection, even among women with normal cytology, increases the risk of CIN2+ and may contribute to bringing the diagnosis forward by more than a decade depending on the case. 11 12 13 14 15 In addition, HPV16/18 coinfection is related to high rates of high-grade lesions (56.7%) and cancer (71.1%), 16 which demonstrates the importance of tests based on the polymerase chain reaction (PCR) technique with genotyping of the viral types.

The association of viral type with the age of the infected woman may also represent an increased risk factor for precursor lesions. Positivity for HPV16 in CIN2+ cases showed an increased risk among English women aged 24 to 44 years old, and for the group aged from 45 to 64 years old, there was a greater association with viral types other than 16/18. 17

Considering the cytological findings, the association of moderate to severe atypia and hrHPV infection among patients referred for colposcopy represents a high risk for histological diagnosis of high-grade and invasive lesions. 18 The presence of severe atypia among women infected with types 16/18 indicates a high risk of CIN2 + , with results varying between 60.2 and 83.6%. 19

Therefore, cytological results, HPV testing, and age have demonstrated their potential in the stratification of risk for precursor lesions and cervical cancer. 18 20 At the Hospital do Câncer de Barretos (HCB, in the Portuguese acronym), all patients performed hr-HPV test in the colposcopy; therefore, the objective of the present study was to stratify the risk of precursor lesions and cancer among women undergoing colposcopy at the Hospital do Câncer de Barretos in São Paulo, state of São Paulo, Brazil.

## Methods

Women ≥ 18 years old who underwent colposcopy at the Cancer Prevention Unit of the HCB from 2017 to 2019 were included. Patients undergoing colposcopy were previously screened by cervical cytology, and HPV tests were performed as a cotest on the same sample of screening cytology or collected during the colposcopy examination. Inconclusive results or findings related to other sites (vagina, vulva, and endometrium) were disregarded.

This was a retrospective cross-sectional study based on a computerized database of the Department of Cancer Prevention of the HCB.

The cervical cancer screening program includes conventional cervical smear and liquid based cytology (LBC, BD SurePath). Samples were sent to the Pathology laboratory for preparation of slides according to the manufacturer's instructions using BD PrepMate and BD PrepStain.

For the detection of HPV DNA, the Cobas HPV Test X480TM (Roche Molecular Systems, Pleasanton, CA, USA) was used. This test detects 14 types of HPV, with HPV 16 and 18 alone and HPV-others encompassing the oncogenic types in a grouping: HPV-31, 33, 35, 39, 45, 51, 52, 56, 58, 59, 66, and 68. 21

Women are referred for colposcopy due to abnormal cervical screening at the HCB. During colposcopy, biopsy is performed when the gynecologist identifies a lesion in the examination. Biopsy was not performed when squamocolumnar junction (SCJ) was visualized and no lesion was identified by the gynecologist. Finally, when SCJ was not visualized and no lesion was identified by the gynecologist, an endocervical curettage (ECC) was performed.

The gold standard considered was the combination of colposcopic examination and anatomopathological results for cases undergoing biopsies or excisional therapeutic procedures (excision of the transformation zone of the cervix [TZE]). The final histological diagnosis was established according to the most severe result obtained.

Analyses of comparisons of proportions were performed using the chi-squared test, considering that the differences between the groups were statistically significant when the *p* -value was < 0.05. A confidence interval (CI) of 95% was considered. IBM SPSS Statistics for Windows version 27 (IBM Corp., Armonk, NY, USA) was used for data storage and statistical analysis.

The present study was conducted in accordance with the guidelines for good clinical practice and was approved by the Ethics Committee of the HCB under CAAE number 50357321.9.0000.5437.

## Results

**Fig. 1:**
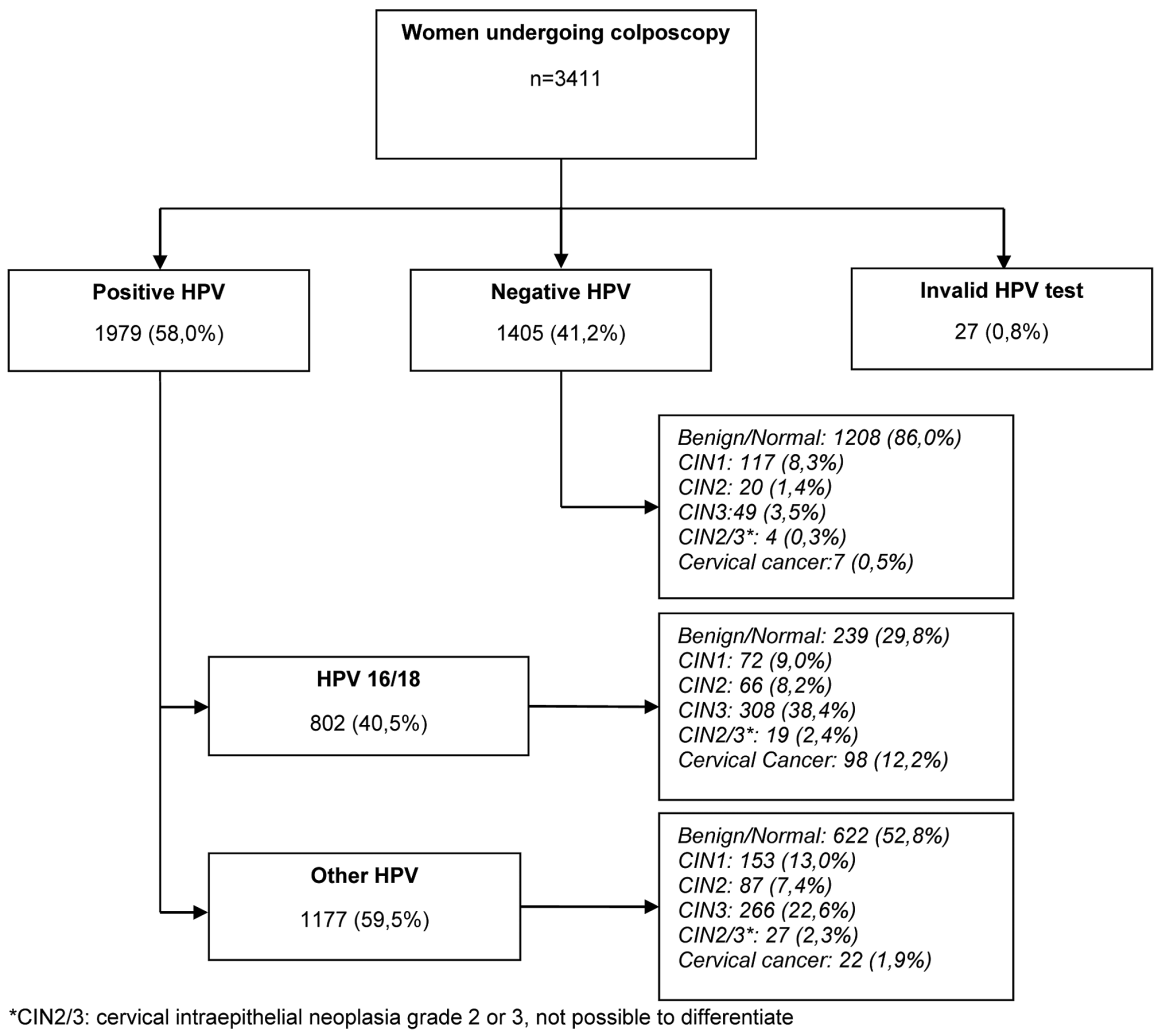
Women undergoing colposcopy in relation to the HPV test and final diagnosis.

A total of 3,411 women aged from 18 to 93 years old (mean 42.5 years old) who underwent colposcopy between March 2017 and December 2019 were included; 27.4% of the participants were tested for HPV at the time of screening (associated with cytology), and 72.6% were tested during colposcopy. The positivity rate for HPV was 58.0% (1,979 women), with 802 positive for HPV16/18 (40.5%) and 1,177 positive for other HPV types (59.5%). HPV16 alone was detected in 19.6% of the cases. Among the women positive for types 16/18, there was a higher prevalence (38.4%) of CIN3. Among the women positive for other viral types than 16/18, 52.8% had no intraepithelial lesions or cancer. In the group of women who tested negative for HPV, 86% had benign findings on colposcopy associated with pathological findings. Our findings show the evidence of false positives in DNA-HPV results since we find ∼ 5% of CIN2+ findings in HPV-negative cases, which could be explained by the possibility of a few cases of cervical cancer not being caused by HPV ( Fig. 1 ).

Regarding cytology, the presence of atypical squamous cells of undetermined significance, which cannot rule out a high-grade lesion (ASC-H), was the most frequent finding (40.8%) and the cytological classification that most commonly pushes the patient to the complementary examination of colposcopy. The mean ages of the women diagnosed with high-grade intraepithelial lesions were 36.4 years old and, for cases of cervical cancer, 43.8 years old ( Table 1 ).

**Table 1:** Study population according to HPV test, age, cytology, and final diagnosis

				Mean age (years old)
			*n* (%)	
HPV genotyping	Positive		1979 (58.0)	40.1
		HPV16	388 (19.6)	38.3
		HPV18	101 (5.1)	40.0
		Other HPV	1177 (59.5)	41.6
		HPV16 and HPV18	12 (0.6)	30.4
		HPV16 and other HPV	214 (10.8)	37.3
		HPV18 and other HPV	68 (3.4)	40.2
		HPV16, HPV18, and other HPV	19 (1.0)	28.4
	Negative		1405 (41.2)	45.7
	Invalid		27 (0.8)	44.9
Cytology	ASC-H		1392 (40.8)	42.3
	AGC		480 (14.1)	43.8
	ASC-US		418 (12.3)	42.8
	HSIL		393 (11.5)	37.9
	LSIL		362 (10.6)	42.0
	NILM		165 (4.8)	45.0
	HSIL (inv)		43 (1.3)	43.9
	Atypical cells of undefined origin		80 (2.3)	46.7
	Endometrial cells a		28 (0.8)	52.7
	Unsatisfactory		24 (0.7)	51.2
	AIS		14 (0.4)	43.1
	SCC		9 (0.3)	49.1
	IA		3 (0.1)	47.7
Final Diagnosis	Benign		2089 (61.2)	45.7
	CIN1		344 (10.1)	38.4
	CIN2		174 (5.1)	35.4
	CIN3		627 (18.4)	36.4
	CIN2/3 b		50 (1.5)	35.6
	Adenocarcinoma in situ		41 (1.2)	39.6
	Invasive adenocarcinoma		7 (0.2)	47.3
	Squamous cell carcinomas		79 (2.3)	43.8
Total			3411 (100)	42.5

Abbreviations: AGC-US, atypical glandular endocervical cells 23 ; AIS, adenocarcinoma in situ; ASC-H, atypical squamous cells cannot exclude high-grade squamous intraepithelial lesion; ASC-US, atypical squamous cells of undetermined significance; HSIL, high grade squamous intraepithelial lesion; HSIL (inv), high grade squamous intraepithelial lesion cannot exclude invasion 22 ; IA, Invasive adenocarcinoma; LSIL, low grade squamous intraepithelial lesion; NILM, negative for intraepithelial lesion or malignancy; SCC, squamous cell carcinoma.

a Endometrial cells (in a woman ≥ 45 years old.

b CIN2/3: cervical intraepithelial neoplasia grade 2 or 3, not possible to differentiate.

Considering the diagnoses of CIN2/3 and cervical cancer, the HPV positivity rates were 90.8% and 94.5%, respectively. Types 16 and 18 were detected in 50.8% of the women with CIN2/3 and in 81.7% of the patients diagnosed with cancer. HSIL and ASCH were the types of cytological atypia most frequently associated with the diagnosis of CIN2+ ( Table 2 ).

**Table 2:** Characterization of the study population according to the final diagnosis

Final diagnosis	Total	Positive HPV	hrHPV genotyping		Cytology						
			HPV 16/18	Other HPV	SCC	IA	AIS	HSIL (inv)	HSIL	ASC-H	Others *
Benign	2089	861 (41.2)	239 (27.8)	622 (72.2)	0 (0.0)	0 (0.0)	0 (0.0)	2 (0.1)	70 (3.4)	822 (39.4)	1195 (57.2)
CIN1	344	225 (65.4)	72 (32.0)	153 (68.0)	0 (0.0)	0 (0.0)	0 (0.0)	0 (0.0)	21 (6.1)	142 (41.3)	181 (52.6)
CIN2/3	851	773 (90.8)	393 (50.8)	380 (49.2)	2 (0.2)	1 (0.1)	1(0.1)	29 (3.4)	266 (31.2)	394 (46.3)	158 (18.6)
Cervical cancer	127	120 (94.5)	98 (81.7)	22 (18.3)	7 (5.5)	2 (1.6)	13 (10.2)	12 (9.5)	36 (28.4)	34 (26.8)	23 (18.1)

Abbreviations: AIS, adenocarcinoma in situ; ASC-H, atypical squamous cells cannot exclude high-grade squamous intraepithelial lesion; HPV, human papillomavirus; HSIL (inv), high grade squamous intraepithelial lesion cannot exclude invasion 22 ; IA, Invasive adenocarcinoma; SCC, squamous cell carcinoma.

* Others: AGC/ASC-US/ Atypical cells of undefined origin /LSIL/endometrial cells/unsatisfactory/NILM.

**Fig. 2:**
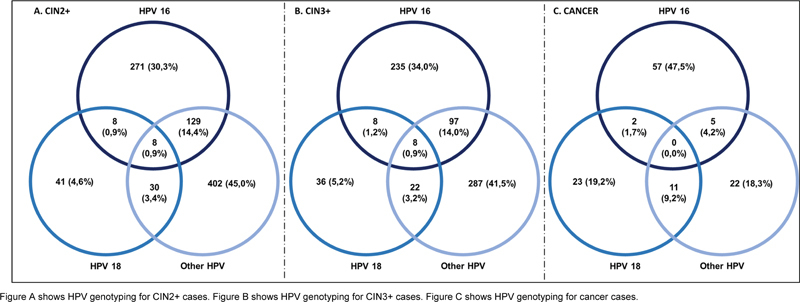
HPV genotyping for CIN2 + , CIN3 + , and cancer.

For the analysis of HPV positivity in the CIN2+ and CIN3+ populations, there was greater positivity in the group of other HPVs (45 and 41.5%, respectively) and HPV16 alone (30.3 and 34%, respectively). The most frequent combination was observed in the combined HPV16 and other HPV groups, namely, 14.4% for CIN2+ and 14.0% for CIN3+ ( Fig. 2 ).

Positivity for HPV 16 and 18 was associated with a higher prevalence of diagnosis of CIN2, CIN3 and cancer (8.4, 39.3, and 12.5%, respectively), while other HPV types and negative HPV showed higher prevalence of diagnosis of ≤ CIN1 (67.4 and 94.6%, respectively). Regarding cytology, the diagnoses of benign and low-grade intraepithelial lesions were more frequent among women with cytology showing ASCUS, LSIL or NILM. CIN2/3 was more frequent among women with AIS, IA, SCC, HSIL (invasive) or HSIL cytology ( Table 3 ).

**Table 3:** Prevalence of HPV genotyping and cytology according to final diagnosis

	≤ CIN1	CIN2	CIN3	Cancer	*p-value*
	n (%; 95%IC)	n (%; 95%IC)	n (%; 95%IC)	n (%; 95%IC)	
hrHPV genotyping					
HPV 16/18	311 (39.7%; 36.3–43.2)	66 (8.4%; 6.6–10.6)	308 (39.3%; 35.9–42.9)	98 (12.5%; 10.3–15.0)	0.00 *
Other HPV	775 (67.4%; 64.6–70.1)	87 (7.6%; 6.1–9.2)	266 (23.1%; 20.7–25.7)	22 (1.9%; 1.2–2.9)	0.00 *
Negative HPV	1325 (94.6%; 93.3–95.7)	20 (1.4%; 0.9–2.2)	49 (3.5%; 2.6–4.6)	7 (0.5%; 0.2–1.0)	0.00 *
Cytology					
ASC-H	964 (70.5%; 68.0–72.9)	97 (7.1%; 5.8–8.6)	273 (20.0%; 17.9–22.2)	34 (2.5%; 1.7–3.5)	0.000 *
AGC	433 (91.2%; 88.2–93.6)	1 (0.2%; 0.0–1.2)	29 (6.1%; 4.1–8.7)	12 (2.5%; 1.3–4.4)	0.000 *
ASC-US	364 (87.3%; 83.7–90.3)	16 (3.8%; 2.2–6.2)	34 (8.2%; 5.7–11.2)	3 (0.7%; 0.2–2.1)	0.000 *
HSIL	91 (24.0%; 19.8–28.6)	34 (9.0%; 6.3–12.3)	218 (57.5%; 52.4–62.6)	36 (9.5%; 6.7–12.9)	0.000 *
LSIL	315 (88.0%; 84.2–91.2)	21 (5.9%; 3.7–8.8)	21 (5.9%; 3.7–8.8)	1 (0.3%; 0.0–1.6)	0.000 *
NILM	150 (91.5%; 86.1–95.3)	3 (1.8%; 0.4–5.3)	10 (6.1%; 3.0–11.0)	1 (0.6%; 0.0–3.6)	0.000 *
Atypical cells of undefined origin	63 (78.8%; 68.2–87.1)	0 (0.0%; 0.0–4.5 ** )	12 (15.0%; 8.0–24.7)	5 (6.3%; 2.1–14.0)	0.085
HSIL (inv)	2 (4.8%; 0.6–16.2)	2 (4.8%; 0.6–16.2)	26 (61.9%; 45.6–76.4)	12 (28.6%; 15.7–44.6)	0.000 *
AIS	0 (0.0%; 0.0–23.2 ** )	0 (0.0%; 0.0–23.2 ** )	1 (7.1%; 0.2–33.9)	13 (92.9%; 66.1–99.8)	0.000 *
SCC	0 (0.0%; 0.0–33.6 ** )	0 (0.0%; 0.0–33.6 ** )	2 (22.2%; 2.8–60.0)	7 (77.8%; 40.0–97.1)	0.000 *
IA	0 (0.0%; 0.0–70.8 ** )	0 (0.0%; 0.0–70.8 ** )	1 (33.3%; 0.8–90.6)	2 (66.7%; 9.4–99.2)	0.002

Abbreviations: AGC-US, atypical glandular endocervical cells 23 ; AIS, adenocarcinoma in situ; ASC-H, atypical squamous cells cannot exclude high-grade squamous intraepithelial lesion; ASC-US, atypical squamous cells of undetermined significance; HSIL, high grade squamous intraepithelial lesion; HSIL (inv), high grade squamous intraepithelial lesion cannot exclude invasion 22 ; IA, Invasive adenocarcinoma; LSIL, low grade squamous intraepithelial lesion; NILM, negative for intraepithelial lesion or malignancy; SCC, squamous cell carcinoma.

* p-value < 0.01.

** One-sided 97.5% confidence interval.

**Fig. 3:**
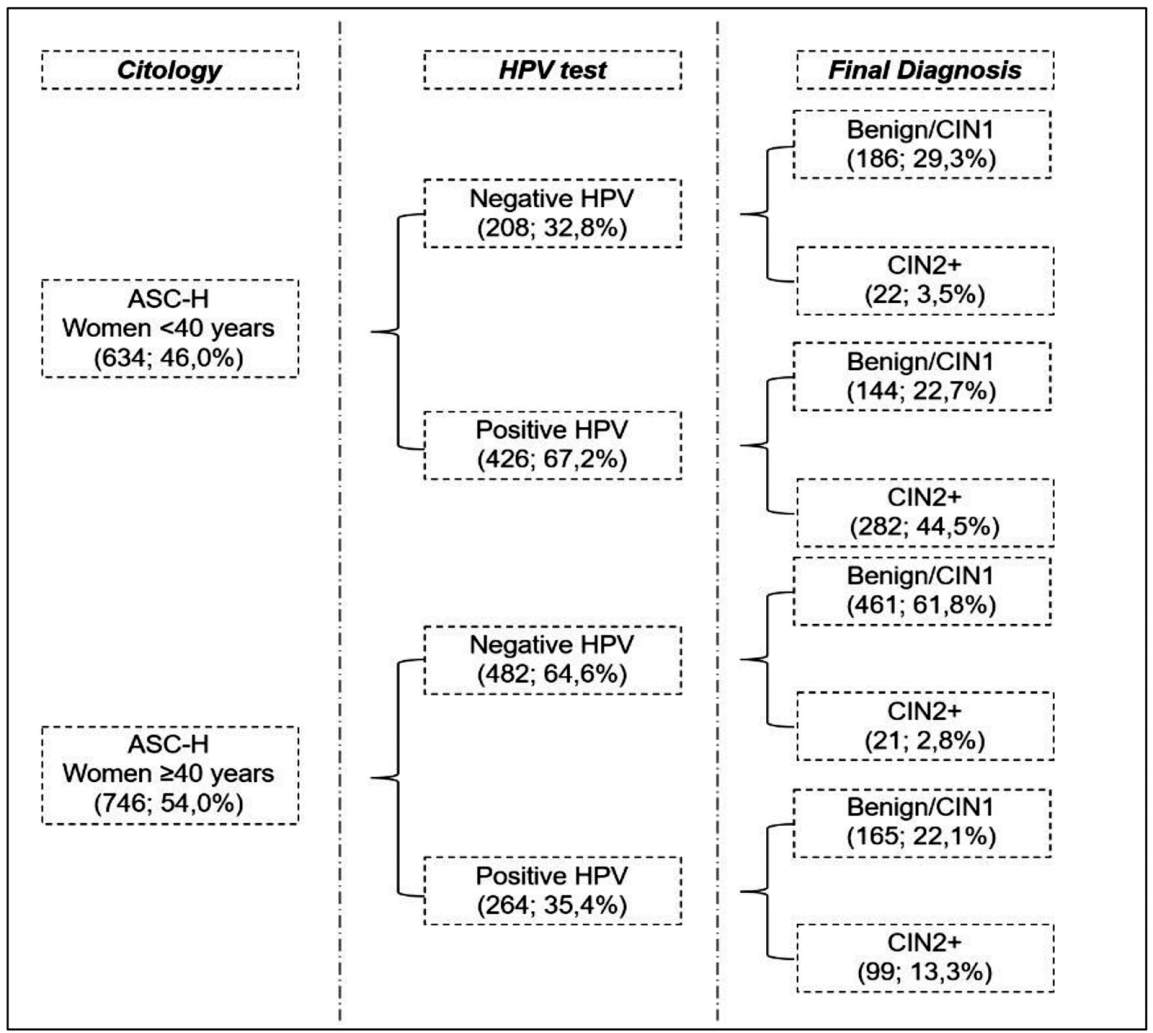
Characterization of patients with ASC-H cytology.

Patients classified as ASCH were the most frequent finding in cytology ( Table 1 ). In total, 54.0% of these patients are ≥ 40 years old. We observe in this group 64.6% negative HPV associated with 61.8% of diagnosis as benign/CIN1. Otherwise, women < 40 years old showed a positivity for HPV in 67,2%. In this group, 44,5% had the diagnosis of CIN2+ ( Fig. 3 ).

**Fig. 4:**
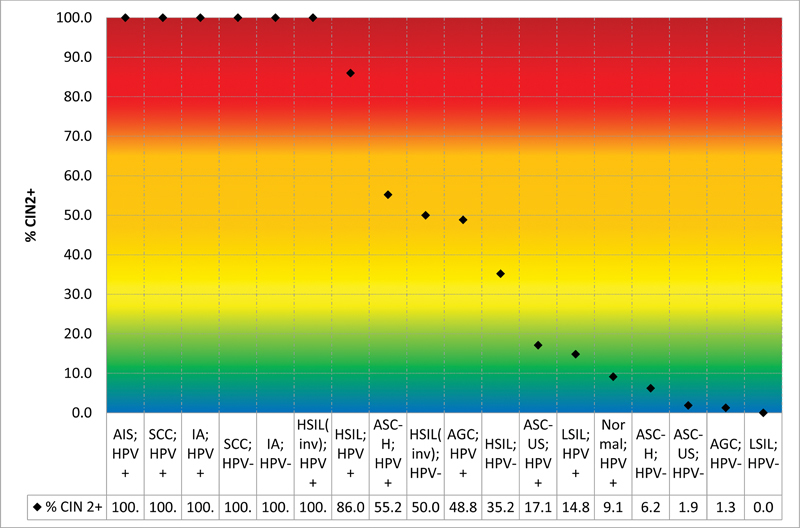
Prevalence of CIN2+ according to cytology and HPV test results.

Figure 4 shows the risk of CIN2+ according to the combination of cytological results and HPV tests.

**Fig. 5:**
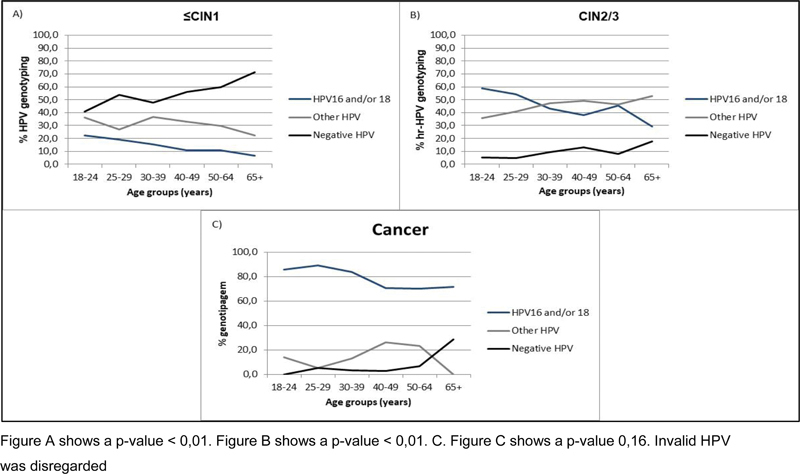
Association between HPV genotype and age at final diagnosis.

There was a higher prevalence of infection by type 16/18 among younger women diagnosed with CIN2/3 and the involvement of the other viral types increased proportionally with increasing age. Among women diagnosed with cancer, a higher incidence of infection by type 16/18 was also found in all age groups, but there was a tendency for other viral types to increase beginning at 35 years old ( Fig. 5 ).

## Discussion

The present study aimed to stratify the risk of precursor lesions and cervical cancer among women who were attended at a reference center for colposcopy, considering age, the cytological test result, and HPV genotype.

The possibility of molecular tests associated with colposcopic and cytological evaluation increases the accuracy of diagnosis, and the high negative predictive value of HPV tests confers a low risk of relevant tissue damage among women who test negative. In our study, 41.19% of the women were negative for hrHPV, of which 86% were negative for intraepithelial lesions and cervical cancer. Other studies have demonstrated the association between colposcopic and histological findings and HPV infection status. 18 20 24 Among women referred for colposcopy, a large group showed cytological results of mild atypia or undetermined findings; for these cases, some groups of low-risk HPV (not identified by the method used in the study) and some results related to tissue repair, inflammation, and atrophy are expected.

In the qualitative evaluation of the composition of viral types among women who tested positive, it was observed that the majority (59.5%) was infected with non-16/18 types. However, there is a clear trend toward benign findings and low-grade intraepithelial lesions in this group of women, whereas type 16/18 increases the risk of CIN3 + . The ESTAMPA study conducted in Latin American countries also found an association between HPV16/18 positivity and the severity of tissue lesions (30.6% CIN2, 56.7% CIN3, and 71.1% cancer). 16 The impossibility of individual genotyping of HPV types other than 16/18 is a limitation of the present study because it could stratify, for example, the importance of types 31, 33, and 45 that have been demonstrated in other publications. 25 26 27 28 29 30

Cytological atypia classified as ASCH was the most frequent, and in 40.8% of the cases, the need for colposcopic examination was justified. Among the women who received this cytological report, 50% tested positive for HPV, and a trend of CIN2/3 diagnoses was observed for the group of younger women. Other studies demonstrated the association of ASCH with precursor lesions and cancer according to HPV status. 19 31 32 33

There is a clear association of ASCH results with factors of atrophy of the cervical epithelium among postmenopausal women, and when associated with negative HPV > 40 years old, they usually represent benign findings in histological investigation.

Women with SCC and AI cytology, regardless of the HPV test status, had a 100% diagnosis of CIN2 +/CIN3 + , which reflects the high specificity of cytology directly associated with the degree of atypia. When associated with positive HPV, the cytology HSIL and ASCH presented a diagnosis of CIN2+ in 86 and 55.2% of patients, respectively. In the absence of HPV infection, CIN2+ detection decreases to < 50% for these same cytological results. These results agree with those of another study that reported a specificity of 86.6% in the diagnosis of CIN2+ among women with HSIL and positive HPV16/18. 18

In view of the risks of developing precursor lesions and cervical cancer, the detection of HPV has shown great benefits, especially due to the increased sensitivity in detecting CIN3 + . 33 34 35 Several studies have implemented the use of HPV tests in population screening of precursor lesions and cervical cancer in place of cytology, which makes it possible to extend the interval of repetition of the exams to periods of 5 years. 36 37 In Brazil, HPV testing is not available for population screening in the public health system, which is still based on cytological tests. 10 Some isolated and regional initiatives have proposed the use of HPV tests with results that endorse its applicability, including positive results in cost-effectiveness evaluations among Brazilian women. 11 38

Our study did not aim to evaluate the primary screening of cervical lesions by molecular tests but focused on the importance of stratifying the risk of women previously screened by cytological tests, considering age and the results of the investigation for HPV by genotyping. The results demonstrate the importance of additional molecular tests, especially among women with cytological tests classified as inconclusive changes and atypia of undetermined significance.

In addition, the possibility of partial HPV genotyping may indicate the need for clinical procedures and personalized follow-up protocols for each patient with individual risk stratification. Although it was not the focus of the present study, the HPV vaccinated populations may also be an additional benefit with HPV genotyping for those viral types that are not protected by the vaccine.

The results described here reinforce the importance of types 16/18 in the carcinogenesis of cervical cancer and add evidence regarding the participation of other viral types in a large proportion of women diagnosed with precursor lesions.

## Conclusion

The highest risks of precursor lesions and cervical cancer were found among women with positive HPV 16/18 tests and severe cytological atypia in population screening tests. Patients with cytology classified as ASCH may have a risk of CIN2+ stratified by the HPV test, and the number of positive tests will be higher among younger women < 40 years old.
